# Effect of Nonsurgical Spinal Decompression on Intensity of Pain and Herniated Disc Volume in Subacute Lumbar Herniated Disc

**DOI:** 10.1155/2022/6343837

**Published:** 2022-09-19

**Authors:** Eunjoo Choi, Ho Young Gil, Jiyoun Ju, Woong Ki Han, Francis Sahngun Nahm, Pyung-Bok Lee

**Affiliations:** ^1^Department of Anesthesiology and Pain Medicine, Seoul National University Bundang Hospital, 82 Gumi-ro 173 Beon-gil, Bundang-gu, Seongnam 13620, Republic of Korea; ^2^Department of Anesthesiology and Pain Medicine, Ajou University, School of Medicine, 164 World Cup-Ro, Yeongtong-Gu, Suwon 16499, Republic of Korea; ^3^Department of Anesthesiology and Pain Medicine, Seoul National University College of Medicine, 101 Daehak-Ro, Jongno-Gu, Seoul 03080, Republic of Korea

## Abstract

**Objective:**

Nonsurgical spinal decompression therapy (NSDT) is a conservative treatment for the lumbosacral herniated intervertebral disc (L-HIVD). This study aimed to evaluate the clinical effectiveness of the NSDT and change in disc volume through magnetic resonance imaging (MRI) in subacute L-HIVD.

**Methods:**

Sixty patients with subacute L-HIVD were randomized into either the decompression group (group D, *n* = 30) or the nondecompression group (group N, *n* = 30). In group D, NSDT was performed ten times in eight weeks. In group N, pseudodecompression therapy (no force) was performed with the same protocol. Lower back and lower leg pain intensities and functional improvements were measured by the visual analog scale and the Korean Oswestry Disability Index (K-ODI). The change in the lumbosacral disc herniation index (HI) was evaluated through a follow-up MRI three months after the therapy.

**Results:**

The lower leg pain intensity in group D was lower than that in group N at two months (*p*=0.028). Additionally, there were significantly lower K-ODI scores in group D at two and three months (*p*=0.023, 0.019) than in group N. The change in HI after the therapy was −27.6 ± 27.5 (%) in group D and −7.1 ± 24.9 (%) in group N, with a significant difference (*p*=0.017). Approximately 26.9% of patients in group D and no patients in group N showed over 50% reduction in HI (*p*=0.031).

**Conclusion:**

NSDT may be a suitable treatment option for conservative treatment of subacute L-HIVD.

## 1. Introduction

Lumbar herniated intervertebral disc (L-HIVD) is a common cause of lower back pain and radiculopathy [1]. L-HIVD, even with massive disc herniation, usually has a favorable clinical course [2]. Surgical treatment may be considered when patients present with neurologic deficits; however, approximately 60–90% of L-HIVD cases are treated with only conservative treatment [3]. Conservative management for L-HIVD includes education, medication, physiotherapy, and epidural steroid injection. Lumbar traction therapy, a type of physiotherapy treatment, may decrease the pain intensity and reduce the size of the herniated disc in L-HIVD [4, 5]. The effects of lumbar traction therapy have been evaluated in various ways, such as by measuring pain relief and improvements in function or by assessing changes in magnetic resonance imaging (MRI) [5–7]. However, the effectiveness of lumbar traction therapy remains questionable because it has various forms (motorized, gravitational, and manual type), and each type has not been properly evaluated through a well-designed randomized controlled study [8–12].

Traction can be delivered manually by the physician via the weight of the patient through a suspension device or by the patient pulling the bars at the head of the table while lying on a table. These types of traction can be difficult to standardize, and the patient may not be able to tolerate the pull force, which may trigger paravertebral muscle contraction and affect efficacy. Nonsurgical spinal decompression therapy (NSDT) has recently been introduced as a type of motorized traction therapy. It involves the use of a device that can fix the upper and lower body on a splitting bed that allows bending, rotating, and stretching, and a computer program can adjust the direction and angle of traction according to the target disc. NSDT, in contrast to conventional traction therapy, can lower the pressure of the nucleus pulposus in the intervertebral disc to < −100 mmHg. The negative pressure in the intervertebral disc is speculated to increase the blood flow for nutrition and regeneration of the disc, though this remains controversial [13–15]. It can also reduce the pressure on the nerve and facet joints by increasing the width of the intervertebral foramen [8, 15, 16]. The difference between NSDT and conventional traction therapy is the relaxation of the back muscles during axial traction with NSDT [4, 17, 18]. Another advantage of NSDT, compared to traditional traction therapy, is that it can steadily increase traction intensity. While the traction intensity increases, the machine receives feedback and simultaneously relaxes the paraspinal muscles. This allows a stronger decompression force to be applied to the patient [17–21]. An important mechanism of traction therapy is the restoration of the herniated mass by increasing the tension of the posterior longitudinal ligament. The axial traction can increase stress on the posterior longitudinal ligament, cause contraction of the muscles around the vertebrae, and increase the internal pressure of the disc [20, 21]. In contrast, NSDT can reduce the stress on the posterior segment of the lumbar spine by relaxing the contracted paraspinal muscles and posterior fibers during traction. A study comparing NSDT with conventional traction therapy showed that NSDT was more effective in reducing pain intensity and improving function [19]. Additionally, NSDT is believed to create a state of zero-gravitation in a targeted herniated disc using a precise computer program [20].

Although there have been several studies comparing the effectiveness of NSDT with other modalities [7, 22], no previous randomized controlled study has evaluated the change in herniated discs after NSDT using MRI. Many previous studies investigated pain reduction as a primary outcome following procedures [23–25]. In this study, the relationship between pain relief and disc volume reduction after NSDT was evaluated.

We hypothesized that using NSDT for subacute lumbar disc herniations would be clinically effective, as determined by pain relief and improvements in the patient's functions. We also hypothesized the volume of the disc herniation would be reduced, as measured by MRI following NSDT.

## 2. Materials and Methods

A prospective randomized controlled study was conducted in the pain clinic of Seoul National University Bundang Hospital. This study was approved by the institutional review board of Seoul National University Bundang Hospital (IRB No. E-1412-278-002) and was registered with the Clinical Research Information Service (Registration No. KCT0002614). This trial was conducted according to the Consolidated Standards of Reporting Trials (CONSORT) guidelines.

### 2.1. Participants

Among the patients with lower back pain and/or radiculopathy of the lower leg who visited the outpatient clinic, participants who met the following criteria were selected from those diagnosed with lumbar disc herniation on MRI. One researcher (not a doctor) verified the suitability again. The researcher then managed the patients' study schedules. All participants were provided with written and verbal information about the trial before obtaining written consent. The inclusion criteria were as follows: (1) 18–60 years old patients, (2) patients with lower back pain and radicular symptoms, (3) patients diagnosed with lumbar disc herniation using MRI, (4) patients with pain duration of four weeks to three months, (5) patients with a visual analog scale (VAS) score of 4 or more, and (6) patients who had not undergone NSDT treatment. The exclusion criteria were as follows: (1) patients with a history of spinal surgery, (2) patients with a neurological deficit that required emergency surgery, (3) patients receiving osteoporosis medications, (4) patients with compressed fracture, and (5) patients with malignant tumors. Prior to registration, a researcher who was not involved in this study assigned individuals to decompression and nondecompression groups according to a computer-generated random list. The list was operated and managed by the researcher, and concealment of allocation was maintained. The experimental group (*n* = 30, decompression group, group D) was treated with decompression treatment, and the control group (*n* = 30, nondecompression group, group N) was treated with pseudo decompression treatment.

### 2.2. Nonsurgical Decompression Therapy

Nonsurgical spinal decompression therapy was performed with Spine MTK-1 (Shinhwa Medical, Busan, Republic of Korea). The NSDT apparatus has built-in air bladders, disc angle pulls adjustments, and harnesses and can increase the distraction force slowly in the latter part of the decompression. Three split table designs were used for reducing friction in the lumbar muscles. The patient lay in a supine position; a chest and shoulder support system controlled the upper body, and a knee rest was used to eliminate pelvic rotation. Each spinal decompression session begins with the patient being fitted with an adjustable lower body and upper body harness. To initiate active treatment, the machine pulled the patient gently on the lower harness while the upper harness remained stationary, thus distracting the patient's spine. A safety button could be pushed at any time by the patient to release all the tension immediately.

### 2.3. NSDT in a Decompression Group

**Figure 1:**
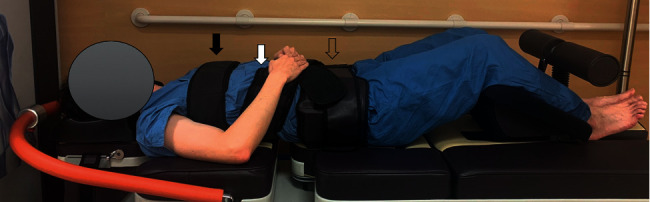
Nonsurgical spinal decompression therapy with Spine MTK-1 (Shinhwa Medical, Busan, Republic of Korea). The position of the patient is supine with flexed knees on the table. The patient is fastened to the table using three belts: the first belt on the chest (black arrow), the second one below the rib cage (white arrow), and the third one on the iliac crest (empty arrow).

Group D received 10 treatment sessions for 30 minutes for eight weeks. The sessions were provided twice a week for the first two weeks and once a week for the remaining six weeks. The distraction force and angle were determined using a computer program based on the patient's body mass and the target disc level. The distraction force was increased by 1 kg per treatment session, starting from half of the body weight minus 5 kg. If the patient complained of pain during treatment, the distraction force was reduced by 25%. Patients laid in the supine position with flexed knees with support on the table. They were fastened to the table using three belts, with the first belt on the chest, the second below the rib cage, and the third on the iliac crest (Figure 1). Decompression therapy was applied with 60 s of hold and 30 s of rest [8, 26, 27]. A safety button could be pushed at any time by the patient to release tension.

### 2.4. NSDT in Nondecompression Group

Group N underwent NSDT using the same protocol and treatment sessions as group D, but no weight loading (distraction force was zero) was applied. If the patient's pain intensity increased by >20% compared to baseline, patients in both groups received nonsteroidal anti-inflammatory drugs and muscle relaxants (Pain intensity was measured by the VAS score, wherein the physician asks the patient to select a point on a line drawn between two ends to express how intensely he/she perceives the pain. The VAS is a continuous scale comprising a horizontal line, usually 100 mm long, anchored by two verbal descriptors (i.e., “no pain” and “worst imaginable pain”)). However, if the patient's pain was not controlled, the patient was prescribed a weak opioid (the second step of the World Health Organization analgesic ladder), such as tramadol. Finally, the patients were excluded if their pain was persistent or aggravated during treatment sessions in both groups.

### 2.5. Clinical Effectiveness

Pain intensity in the lower back and lower extremities with respect to the VAS was measured before NSDT (baseline) and 1, 2, and 3 months after the end of the last session of NSDT. Additionally, the Korean Oswestry Disability Index (K-ODI) was employed for evaluating the degree of disability at the same time point. Information regarding age, sex, height, body mass, duration of symptoms, and diagnosis was obtained from each patient.

### 2.6. Measurement of Herniated Disc through MRI

**Figure 2:**
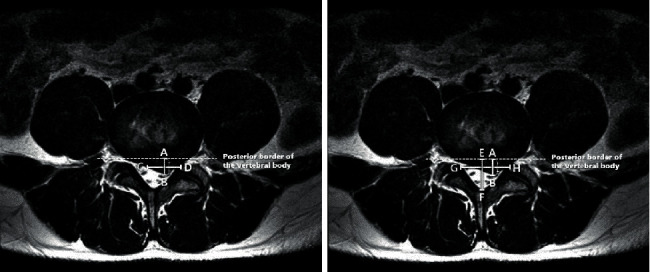
Methods for calculating the herniation index (HI) in the axial plane of the magnetic resonance imaging (MRI) scan. AB: the maximal anteroposterior disc length, which is the diameter of the herniated disc material extended maximally from the posterior border of the vertebral body. CD: the width of the herniated disc material at the level of the middle AB distance of the herniated disc material from the coronal plane of the MRI scan. EF: the maximal anteroposterior canal length. GH: mid-AB distance. HI was calculated using the following formula:

Magnetic resonance imaging was performed before NSDT and three months after the end of all sessions to determine the change in herniated disc following NSDT. The type of HIVD (disc degeneration, prolapse, extrusion, sequestration) and the herniation index (HI) were analyzed. T2-weighted axial images were used for calculating the HI of the disc. The HI was measured on the axial plane with maximal herniation of the intervertebral disc on MRI (Figure 2). MR images were analyzed by two experienced pain clinicians, who were not involved in this study.


(1) $$\begin{array}{c} \frac{\left(\text{AB}\right)\times \left(\text{CD}\right)}{\left(\text{EF}\right)\times \left(\text{GH}\right)}\times 1000. \end{array}$$

The maximal anteroposterior disc length (AB, mm), which is the sagittal distance of the herniated disc material extended maximally from the posterior border of the vertebral body, was measured. The width of the herniated disc material at the level of the middle AB distance (CD, mm) of the herniated disc material from the coronal plane of the MRI, the maximal anteroposterior canal length (EF, mm), and mid-AB distance (GH, mm) were also measured. The HI was calculated using the following formula [28–30]:

(2) $$\begin{array}{c} \text{HI}=\frac{\left(\text{AB}\right)\times \left(\text{CD}\right)}{\left(\text{EF}\right)\times \left(\text{GH}\right)}\times 1000. \end{array}$$

Increased HI indicated a larger volume of disc herniation.

### 2.7. Statistical Analyses

A total sample size of 54 achieved an effect size of 0.55 and 80% power with a type 1 error of 0.05. The effect size was calculated based on the assumption that 50% of the patients in group D would have a 50% reduction in pain intensity. To allow for a 10% dropout rate, the final sample size was 30 patients per group. All measurements are expressed as the mean ± standard deviation or standard error of the mean (%). Patients' age, height, weight, symptom duration, and HI change rate (%) after treatment were compared using the *t*-test or Mann–Whitney *U* test. For cases in which a significant time-dependent change in pain intensity (VAS) and K-ODI occurred within the same group, a repeated-measures analysis of variance was performed. Additionally, logistic regression was performed to calculate the adjusted odds ratio with a 95% confidence interval for identifying patient factors associated with a successful NSDT. The Hosmer–Lemeshow goodness of fit was used for testing the estimated logistic regression model. All statistical analyses were performed using the SPSS statistics program version 21.0 (IBM Corp, Armonk, NY, USA). A *p* value of <0.05 was considered statistically significant.

## 3. Results

**Figure 3:**
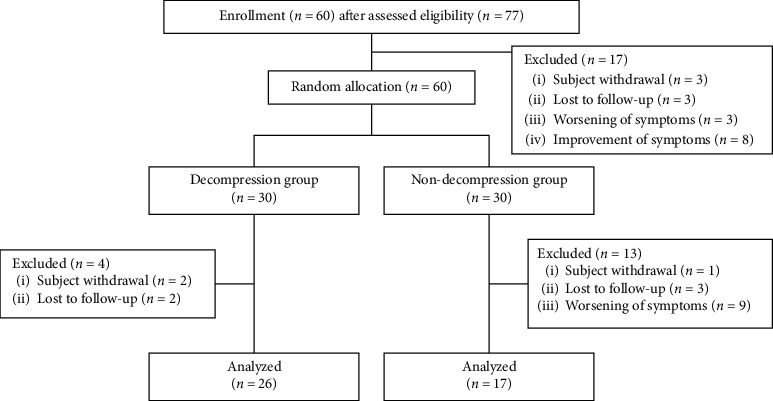
CONSORT diagram of patients enrolled in the study.

Among the 77 patients screened for eligibility, 60 patients were randomized to either group D (*n* = 30) or group N (*n* = 30) (Figure 3). Four patients in group D (two patients refused procedures and others were lost to follow up) and 13 patients in group N (one patient refused procedures, three were lost to follow up, and others had worsening symptoms) were excluded. Thus, data from 43 patients (26 in group D and 17 in group N) were included in the final analysis.

The demographics and clinical variables of the patients are presented in Table 1. The central type of L-HIVD was the most common in both groups, with no significant difference. Other variables showed no significant difference between the two groups.

**Figure 4:**
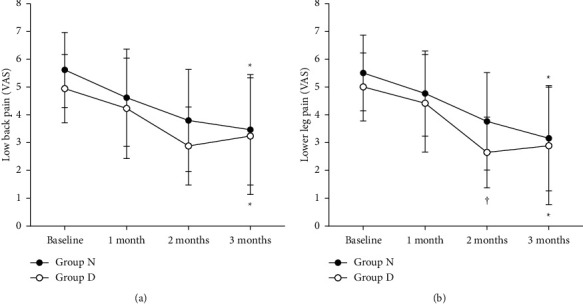
Changes in visual analog scale (VAS) scores of lower back pain (a) and lower leg pain intensity (b) between group D and group N Both groups showed a decrease in lower back pain intensity from baseline to 3 months (*p* < 0.001). However, lower back pain intensity was not significantly different between the groups at any time. The lower leg pain intensity showed a significant decrease in VAS scores from baseline to 3 months (*p* < 0.001), and the lower leg pain intensity in group D showed a significantly lower VAS score than that in group N at 2 months (*p*=0.028). The error bar indicates the standard deviation. ^*∗*^Significant at *p* < 0.001 when compared to the baseline VAS score. ^†^Significant at *p* < 0.001 between D and N groups.

**Figure 5:**
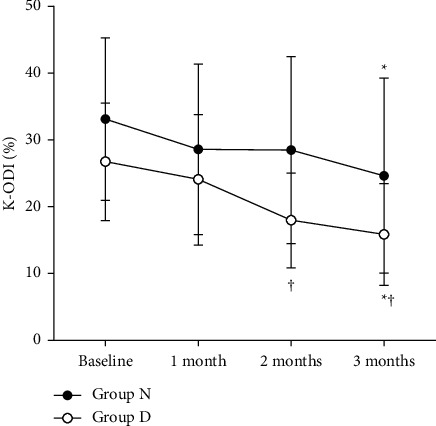
Changes in the Korean Oswestry Disability Index (K-ODI) between groups D and N. The K-ODI significantly decreased in both the groups at 3 months compared to baseline (*p* < 0.001). There were significantly lower scores of K-ODI in group D at 2 months (*p*=0.023) and 3 months (*p*=0.019) than in group N. The error bar indicates the standard deviation. ^*∗*^Significant at *p* < 0.001 when compared to the baseline visual analog scale score. ^†^Significant differences between the D and N groups.

Both groups exhibited a significant decrease in the VAS scores for lower back pain intensity from baseline to three months (*p* < 0.001) (Figure 4(a)). However, there were no significant differences in VAS scores between groups D and N at any time point during the follow-up period. The lower leg pain intensity showed a significant decrease in VAS scores from baseline to three months (*p* < 0.001) (Figure 4(b)), and the lower leg pain intensity in group D was lower than that in group N at two months only (*p*=0.028). K-ODI significantly decreased in both groups at three months compared to the baseline (*p* < 0.001). Additionally, there were significantly lower scores of K-ODI in group D at two months (*p*=0.023) and three months (*p*=0.019) (Figure 5) than in group N. None of the patients in either group experienced any adverse event related to NSDT during the follow up period.

The difference in baseline HI was not significant (*p*=0.295, Table 2), and the HI of group D after NSDT was significantly less than that of group N (*p*=0.007). The change in HI after the procedure was −27.6 ± 27.5 (%) in group D and −7.1 ± 24.9 (%) in group N, with a significant difference (*p*=0.017). Approximately 26.9% of patients in group D and none of the patients in group N showed over 50% reduction in HI (*p*=0.031).

## 4. Discussion

These results demonstrated that NSDT with a newly introduced device for subacute L-HIVD significantly reduced the size of the herniated disc, as observed during MRI examination, and contributed to the improvement in leg pain intensity at two months and in function at three months. This randomized controlled trial added to previous research by investigating whether actual decompression worked for subacute L-HIVD and using MRI examination as a measuring tool. This might be the first study to investigate the effect of NSDT on the pain score and herniated disc volume.

Subacute L-HIVD and the associated pain often recover spontaneously [31–35], while nerve edema and fibrous tissue around the L-HIVD decrease over weeks or months. Therefore, it was challenging to identify the therapeutic effects, such as pain relief and reduction of disc volume on subacute L-HIVD by comparing with the control group. This randomized control study is meaningful because the decompression power of NSDT itself contributed to the recovery of subacute L-HIVD.

In this study, the HI calculated based on MRI findings was used, which proved to be a reliable index comparing the volume of the herniated disc after the intervention. All changes in HI in group D (−27.6% ± 27.5%) showed approximately a 30% decrease following NSDT, with a significant difference (*p*=0.017) when compared with group N (−7.1% ± 24.9%). Additionally, there were patients (*n* = 7/26, 26.9%) with a decrease of >50% in HI in group D. In a previous study, segmental traction therapy with physiotherapy showed an effective reduction of herniated mass size as observed using MRI examination [7]. Previous research was not based on applying only NSDT, and the measure of disc volume was also different from that in our study. It can be stated that our study accurately reflects the therapeutic effect of NSDT alone.

There were no significant differences in the relief of lower back pain between groups D and N at any time points during the follow-up period, and the lower leg pain intensity in group D was lower than that of group N at two months. Although there were significantly lower scores of K-ODI in group D at two and three months, this result does not indicate complete improvement of group D compared to that of group N. Although the size of the disc was further reduced, as observed in the MRI examination, and functionally improved in group D, the decrease in the intensity of lower back or leg pain showed little or no difference between the two groups. In a recent meta-analysis, lumbar traction exhibited significant pain reduction and functional improvements only in the short-term [36]. In this study, similar results for functional improvements were observed from two to three months; however, the long-term effect of NSDT could not be confirmed. Although lower back and leg pain intensities significantly decreased in both groups at three months, a comparison of the two groups showed no significant difference. The reason may be found in the natural courses of L-HIVD. Although the principle of reabsorption of the L-HIVD is unclear, the nucleus pulposus of the disc is exposed to the vascular tissue in the epidural space, and the chemokines secreted from macrophages play an important role in phagocytosis. Shrinkage of L-HIVD is caused by decreased nutrient supply [34, 37]. It is expected that this natural course of L-HIVD was actively conducted in the acute/subacute phase, and our study was performed on subacute L-HIVD patients (4 weeks–3 months). Therefore, both groups would be expected to recover spontaneously [34, 35, 38], and the difference in pain relief may be minimal.

A previous study analyzed the MRI scans of 32 patients with L-HIVD, and the mean disc volume reduction was 64% (range, 31–78%) during an average period of 13.2 months (range, 3–42 months) [39]. In another study, 66 (67.3%) of a total of 98 patients did not undergo surgery, even in sequestered L-HIVD. Following the MRI scan conducted in 80 patients 6 months later, 6 (10.9%) patients were included in the nonregression, 22 (40%) in the partial regression, and 27 (49.1%) in the complete resolution groups [34]. In our study, group D showed an approximately 30% reduction in HI at three months. It is thought that applying NSDT for L-HIVD in subacute periods can reduce the disc volume more quickly and contribute to the partial improvement in pain relief and function. Additionally, the surgical treatment was superior to the nonsurgical treatment in the acute phase, but there was no significant difference in the reduction of pain and recovery of neurological deficit thereafter [35]. It may be advisable to apply NSDT for patients with L-HIVD in the subacute phase, considering the cost and risk of surgery [40]. Since pain relief and functional improvement in subacute L-HIVD cannot be achieved using NSDT alone, other conservation therapies (analgesics, physiotherapy, exercise, etc.) should be incorporated if necessary.

There were several limitations to this study. First, only patients between 18 and 60 years old were included. Therefore, this result does not apply to children and older patients and needs further research. Second, group N (13/30, 43.3%) had a significantly higher dropout rate than group D (4/30, 13.3%). Some patients in group N (9/13, 30.8%) withdrew after the first or second session owing to worsening symptoms. After the end of the study, the reasons were analyzed after interviewing the patients on the phone during the follow-up period. Aggravating pain was the first cause, and the pain medication prescription violated the study protocol. Some patients believed that they were allocated to the control group because they did not experience any pain relief and therefore canceled the follow-up. Third, previous studies included more sessions of traction therapy over a longer period [36, 41]. However, the period of our study was somewhat shorter (three months) and the number of treatment sessions was relatively small. If we had set a longer period and conducted more treatment sessions, we might have observed a more meaningful change in L-HIVD. Fourth, it is possible that the controls were not perfectly blinded. It may have been better to apply a slight traction force to patients in the control group, but there is no clear protocol on how much force should be applied. Due to the limitations of this study, better designed studies are required in the future.

## 5. Conclusions

In summary, subacute L-HIVD has a natural recovery course; however, pain can be severe and may require multidisciplinary treatment. A strength of our study was that NSDT significantly reduced the HI calculated based on follow-up MRI examinations. Additionally, patients who received NSDT had partial improvement in pain relief and function. NSDT may be a suitable treatment option for conservative treatment of subacute lumbosacral herniated intervertebral disc.

## Figures and Tables

**Table 1:** Comparison of demographic and clinical variables between the decompression and nondecompression groups.

	Decompression group (*n* = 26)	Nondecompression group (*n* = 17)	*p* value
Age	40.3 ± 11.5	47.4 ± 8.9	0.149
Sex, Male/Female (%)	11/15 (42.3/57.7)	10/7 (58.8/41.2)	0.358
Height (cm)	165.8 ± 9.5	168.5 ± 8.3	0.630
Body mass (kg)	69.2 ± 13.2	69.5 ± 11.1	0.658
Symptom duration (weeks)	7.6 ± 2.6	7.7 ± 2.1	0.906

Level of L-HIVD			0.133
L4–5	12	12	
L5–S1	14	5	

Type of L-HIVD			0.516
Central	20	10	
Paracentral	2	3	
Foraminal	1	2	
Mixed	3	2	

^*∗*^ *p* < 0.05. L-HIVD, lumbar herniated intervertebral disc.

**Table 2:** Comparison of demographic and clinical variables between the decompression and nondecompression groups.

	Decompression group (*n* = 26)	Nondecompression group (*n* = 17)	*p* value
Baseline HI	348.6 ± 183.1	412.4 ± 206.8	0.295
Post HI	232.1 ± 130.3	369.1 ± 186.1	0.007^*∗*^
Change in HI (%)	−27.6 ± 27.5	−7.1 ± 24.9	0.017^*∗*^
≥30% of reduction, *n* (%)	11 (42.3)	3 (17.6)	0.086
≤30% of reduction, *n* (%)	15 (57.7)	14 (82.4)	
≥50% of reduction, *n* (%)	7 (26.9)	0 (0)	0.031^*∗*^
≤50% of reduction, *n* (%)	19 (73.1)	17 (100)	

Data are reported as the mean ± standard deviation or number of patients. ^*∗*^*p* < 0.05. HI, herniation index.

## Data Availability

The datasets used and/or analyzed during this study are available from the corresponding author upon reasonable request.
